# Coleoptera of Canada

**DOI:** 10.3897/zookeys.819.24724

**Published:** 2019-01-24

**Authors:** Adam J. Brunke, Patrice Bouchard, Hume B. Douglas, Mikko Pentinsaari

**Affiliations:** 1 Agriculture and Agri-Food Canada, Canadian National Collection of Insects, Arachnids and Nematodes, 960 Carling Avenue, Ottawa, Ontario, K1A 0C6, Canada Agriculture and Agri-Food Canada Ottawa Canada; 2 Centre for Biodiversity Genomics, 50 Stone Road East University of Guelph, Guelph, Ontario, N1G 2W1, Canada University of Guelph Guelph Canada

**Keywords:** beetles, biodiversity assessment, Biota of Canada, Coleoptera

## Abstract

The beetle fauna of Canada was assessed, including estimates of yet unreported diversity using information from taxonomists and COI sequence clusters in a BOLD (Barcode of Life Datasystems) COI dataset comprising over 77,000 Canadian records. To date, 8302 species of Coleoptera have been recorded in Canada, a 23% increase from the first assessment in 1979. A total of 639 non-native beetle species have become established in Canada, with most species in the Staphylinidae (153 spp.), Curculionidae (107 spp.), Chrysomelidae (56 spp.) and Carabidae (55 spp.). Based on estimates from the taxonomic community and our BOLD dataset, we estimate that slightly more than 1000 beetle species remain to be reported from Canada, either as new records or undescribed species. Renewed enthusiasm toward and financial support for surveys, especially in the central and western provinces of Canada will be critical for detecting, documenting and describing these species. The Barcode of Life database is still far from comprehensive for Canadian Coleoptera but substantial progress has been made and the number of Barcode Index Numbers (BINs) (as candidate species) has reached nearly 70% of the number of species reported from Canada. Comparison of BINs to observed species in a group of Canadian Staphylinidae suggests that BINs may provide a good estimate of species diversity within the beetles. Histeridae is a diverse family in Canada that is notably underrepresented in BOLD. Families such as Mordellidae, Scraptiidae, Latridiidae, Ptiliidae and Scirtidae are poorly known taxonomically in Canada and are represented in our BOLD dataset by many more BINs than recorded species.

Campbell et al. (1979) provided the first thorough assessment of the biology and diversity of Canadian beetles. That important contribution, based on unpublished lists of Canadian beetle species, was followed by two checklists of Canadian beetle species (Bousquet 1991, Bousquet et al. 2013) that form the foundation of the results presented below. New Canadian records published since Bousquet et al. (2013) are listed in Table 1 under the respective families. Beetle classification has changed significantly over recent decades and continues to improve based on results of phylogenetic analyses of ever-larger datasets. Generally, we follow the classification used in Bousquet et al. (2013) with the following changes: Georissidae, Helophoridae and Hydrochidae separate from Hydrophilidae (Short and Fikáček 2013); Biphyllidae and Byturidae as Cleroidea (Robertson et al. 2015); Cybocephalidae distinct from Nitidulidae (Cline et al. 2014); cerylonid series families as superfamily Coccinelloidea (Robertson et al. 2015); Murmidiidae and Euxestidae distinct from Cerylonidae (Robertson et al. 2015); Teredidae distinct from Bothrideridae (Robertson et al. 2015); Anamorphidae and Mycetaeidae distinct from Endomychidae (Robertson et al. 2015); Cimberididae distinct from Nemonychidae (Shin et al. 2018).

Coleopterists within the taxonomic community were asked for estimates of undescribed and unreported Canadian beetles in their group of specialisation (contributors listed in Acknowledgments). Estimates accounted for both unrecognised distribution records and undescribed species, including those indicated by BINs (see below). In cases of multiple estimates, a range was reported to show the minimum and maximum values. We stress that these values were not intended to be precise but were included to provide the reader with an estimate of how well each group is known taxonomically in Canada. A dataset comprised of 77,626 Canadian Coleoptera records associated with a BIN (Barcode Index Number, Ratnasingham and Hebert (2013)) in BOLD (Barcode of Life Datasystems) was also used to estimate beetle diversity in Canada. Number of BINs was used as a proxy for species diversity in Canada with the caveat that there will be instances where closely related species may share a BIN or a single species may be represented by multiple BINs. Beetle families with fewer reported species than BINs were estimated to contain in Canada at least as many undescribed or unreported species as BINs. Families with many more described species than BINs are considered to be underrepresented in BOLD and would benefit from focused sequencing and collecting effort in the future.

Canadian beetles are classified in the suborders Archostemata, Adephaga, and Polyphaga (Table 1). Currently, 8302 species have been recorded in Canada (Table 1), a 23% increase from 6742 in 1979, 13% from 7326 in 1991 and 1.8% from 8149 in 2013). The number of Canadian species in the families Anthicidae, Clambidae, Corylophidae, Hydraenidae, Leiodidae, Psephenidae, Ptiliidae, and Scirtidae have more than doubled since 1979 (Table 1). The four most diverse families of beetles in Canada are the Staphylinidae (1774 spp.), Carabidae (983 spp.), Curculionidae (826 spp.) and Chrysomelidae (595 spp.) (Table 1). Of these, the number of Canadian Staphylinidae has increased most since 1979 (by 840 species, 90%) and the total number of species in Canada might eventually exceed 2000 (Table 1). The number of BINs in the BOLD database (Table 1) for Canadian Coleoptera is nearly 70% of the number of known beetle species for Canada. All of the higher groups of Canadian beetles have associated BINs except for the polyphagan superfamily Dascilloidea, with the single Canadian species *Sandalusniger* Knoch.

Although our knowledge of Canadian beetle diversity has steadily increased between 1979, 1991, 2013 and 2018, significant contributions can still be made in each province and territory as sampling has been far from exhaustive (for overall estimates of undescribed or unrecorded beetle species, see Table 1). Most biomes in Canada are still only superficially sampled, especially those in central and western Canada. Despite much recent survey work over the past 15 years, more than 300 species were added to the provincial beetle fauna of New Brunswick only two years ago (Webster et al. 2016a). Continued survey work, using a variety of collection techniques, will be necessary for Canada to respond to important changes to its dynamic fauna, such as new invasive species and thermophilic species expanding their range northward in response to global climate change.

In total, 639 non-native beetle species are established in Canada (Table 1), although some of these may eventually be proven to be naturally Holarctic. While a few were introduced intentionally for the biological control of weeds and insects (e.g., De Clerck-Floate and Cárcamo 2011), most have been introduced into North America accidentally through various pathways including dry ballast, wood packing material, and agricultural and horticultural commodities such as stored grain, moss and plant stock (e.g., Klimaszewski and Brunke 2018). The families with the highest number of non-native species in Canada are Staphylinidae (153 spp.), Curculionidae (107 spp.), Chrysomelidae (56 spp.), and Carabidae (55 spp.).

Nineteen beetle families are currently not or only poorly represented in BOLD by Canadian specimens, i.e., the number of BINs is <20% of the number of recorded species in Canada, making it difficult to use barcode data to assess overall taxonomic knowledge (Table 1). These families typically contain few known species in Canada based on published taxonomic data (Table 1). Sixteen of these families are not represented in BOLD by Canadian specimens: Micromalthidae (Archostemata); Georissidae and Sphaeritidae (Hydrophiloidea); Glaphyridae and Passalidae (Scarabaeoidea); Rhipiceridae (Dascilloidea); Dryopidae and Limnichidae (Byrrhoidea); Nosodendridae (Derodontoidea); Endecatomidae (Bostrichoidea); Biphyllidae (Cleroidea); Prostomidae (Tenebrionoidea); Bothrideridae, Euxestidae, Mycetaeidae, and Teredidae (Coccinelloidea). Efforts are underway to generate DNA barcodes for these families based on Canadian specimens. The family Histeridae, which has more than 130 species in Canada, is particularly underrepresented, with only 22 BINs (i.e., approximately 16% of the known diversity) currently available in BOLD. Most members of this family are small, and live in microhabitats that are not sampled frequently such as mammal and bird nests, or under bark (Bousquet and Laplante 2006). This lack of representation could also be partly due to sequencing bias against Histeridae resulting from primer mismatch, or differences in DNA preservation at the collecting and archiving stages. For example, only 3% (8/256) of a diverse sample of Histeridae specimens from the Canadian National Collection of Insects, Arachnids and Nematodes (CNC, Agriculture and Agri-Food Canada) yielded barcode-compliant (and therefore BIN-compatible) sequences, versus 22% of submitted CNCStaphylinidae (522/2356).

Based on the number of BINs in BOLD for Canadian specimens, sixteen beetle families are more diverse in Canada than would appear from the recorded number of species (Table 1). The families where the number of BINs most greatly exceeds the number of species reported in Canada are: Mordellidae (+32 BINs) and Scraptiidae (+28 BINs) (Tenebrionoidea), Latridiidae (+23 BINs) (Coccinelloidea), Ptiliidae (+21 BINs) (Staphylinoidea), and Scirtidae (+21 BINs) (Scirtoidea). These families, generally with poorly known and small-sized species, require focused taxonomic studies because they may contain many undescribed species or described species yet unreported from Canada. This work should reconcile the unidentified BIN clusters with available names and describe any species new to science to adequately document the Canadian fauna. Researchers at the CNC and the Canadian Museum of Nature have made numerous contributions to the knowledge of Canadian Coleoptera. However, because most federal employees in Canada focus their research on agriculturally-significant taxa (see Bouchard et al. (2017) for plant-feeding taxa with high economic concern in Canada and in agroecosystems of our trading partners), beetle groups without either plant pests or well-known beneficial species have been given a lower taxonomic research priority. Canadian universities have until recently included taxonomic research on non-economically important beetles, although they currently support a minute fraction of research on Canadian Coleoptera.

The total estimated number of undescribed and unreported beetle species for Canada is 1080 to 1280 species (Table 1) based on expert estimates and species predicted by BINs including Canadian specimens in BOLD. The beetle families with the greatest number of taxonomist-estimated unrecognised diversity in Canada include the Staphylinidae, Carabidae, Ptiliidae, Curculionidae and Chrysomelidae, most of which include either plant pests or beneficial predators and parasitoids. These numbers represent the best available estimate of unrecorded Coleoptera diversity, although they must be interpreted with respect to limitations of expert opinion, BOLD database sampling, potential inaccuracies of the most current checklist (Bousquet et al. 2013), and BIN calculation methods. While we expect the exact numbers to change with further taxonomic research, the general trends reported herein should not.

**Table 1.** Census of Coleoptera in Canada. Information sources refer to those available since the publication of Bousquet et al. (2013).

Taxon^1^	No. species reported in Campbell et al. (1979)	No. species currently known from Canada^2^	No. BINs^3^ available for Canadian species	Est. no. undescribed or unrecorded in Canada	General distribution by ecozone^3A^	Information sources
**Suborder Archostemata**						
Cupedidae	3	3	3	0	Mixedwood Plains, Montane Cordillera	
Micromalthidae	1	1	0	0	Pacific Maritime	
**Suborder Adephaga**						
Amphizoidae	3	3	2	0	Pacific Maritime, Boreal Cordillera, Montane Cordillera	
Carabidae	861^4^	983 (55)	652	150	all ecozones	Lewis et al. 2015
Dytiscidae	285	280	181	7	all ecozones	
Gyrinidae	30	34	29	0	all except Arctic	
Haliplidae	38	35	20	0	southern Arctic and southward	van Vondel and Alarie 2016
Noteridae	0	2	1	0	Mixedwood Plains	
Rhysodidae	?^4^	2	1	0	Pacific Maritime, Mixedwood Plains	
Trachypachidae	?^4^	2	2	0	Boreal Plains, Mountain Cordillera	
**Suborder Polyphaga**						
**Superfamily Scirtoidea**						
Eucinetidae	5	7 (1)	6	2	Boreal ecozones and southward	
Clambidae	3	7 (2)	6	0	Boreal ecozones and southward	
Scirtidae ^5^	12	25 (1)	46	21	Taiga ecozones and southward	
**Superfamily Hydrophiloidea**						
Histeridae	87	137 (12)	22	11	all except Arctic	Brousseau et al. 2014
Georissidae	1	1	0	0	Montane Cordillera, Prairies	
Helophoridae	?^6^	27 (1)	22	0	southern Arctic and southward	
Hydrochidae	?^6^	8	4	0	Boreal ecozones and southward	
Hydrophilidae	180^6^	113 (18)	89	3	southern Arctic and southward	
Sphaeritidae	1	1	0	0	Pacific Maritime, Montane Cordillera, Western Interior Basin	
**Superfamily Staphylinoidea**						
Agyrtidae	?^7^	7	2	0	Cordilleras and Mixedwood Plains	
Hydraenidae	13	27	8	0	all except Arctic	
Leiodidae	83^8^	181 (2)	131	15	all except Arctic	Peck and Newton 2017
Ptiliidae	20	48 (12)	69	75	all except Arctic	
Silphidae	27^7^	27 (1)	21	2	all except Arctic	Sikes et al. 2016
Staphylinidae	934^9^	1774 (153)	1135	370	all ecozones	Klimaszewski et al. 2018, A Davies pers. comm.
**Superfamily Scarabaeoidea**						
Geotrupidae	?^10^	12 (1)	10	2	all ecozones south of boreal	
Glaphyridae	?^10^	1	0	1	Pacific Maritime	
Glaresidae	?^10^	2	1	3	Montane Cordillera, Prairies	
Hybosoridae	?^10^	1	1	0	Mixedwood Plains	
**Superfamily Scarabaeoidea**						
Lucanidae	10	14	10	2	southern Taiga ecozones, Hudson Plains and southward	
Ochodaeidae	?^10^	4	2	0	Montane Cordillera, Prairies, Mixedwood Plains	
Passalidae	1	1	0	0	Mixedwood Plains, Prairies	
Scarabaeidae	210^10^	220 (23)	173	24	all except Arctic	
Trogidae	?^10^	15	6	2	south of Taiga ecozones and Boreal Cordillera	
**Superfamily Dascilloidea**						
Rhipiceridae	1	1	0	0	Mixedwood Plains	
**Superfamily Buprestoidea**						
Buprestidae	200	178 (6)	128	6–18	southern Arctic and southward	Lyons et al. 2014
**Superfamily Byrrhoidea**						
Byrrhidae	31	26 (3)	25	3	southern Arctic and southward	
Dryopidae	3	6 (1)	0	0	Boreal ecozones and southward	
Elmidae	16	32	14	0	Hudson Plains, Boreal ecozones and southward	
Heteroceridae	16	28	9	0	Hudson Plains, Boreal ecozones and southward	
**Superfamily Byrrhoidea**						
Limnichidae	5	3	0	0	Boreal Plains, Boreal Shield, Mixedwood Plains	
Lutrochidae	0	1	1	0	Mixedwood Plains	
Psephenidae	1	4	3	0	Mixedwood Plains, Atlantic Maritime	
Ptilodactylidae	2	4	8	4	south of Boreal in the west, Boreal Shield, Mixedwood Plains	
**Superfamily Elateroidea**						
Artematopodidae	4	5	5	0	south of Boreal in the west, Boreal Shield, Mixedwood Plains	
Cantharidae	111	151 (3)	103	5	southern Arctic and southward	
Elateridae	350	385 (7)	302	20–58	southern Arctic and southward	Webster et al. 2016b
Eucnemidae	30	39	27	4	Boreal ecozones and southward	Webster et al. 2016b
Lampyridae	26	32 (1)	31	2	Hudson Plains, Boreal and southward	
Lycidae	23	29	37	8	Boreal ecozones and southward	
Phengodidae	2	1	2	1	Mixedwood Plains	
Throscidae	8	8	19	11	Boreal ecozones and southward	
**Superfamily Derodontoidea**						
Derodontidae	6	8 (1)	4	0	Boreal ecozones and southward	
Nosodendridae	1	2	0	0	Montane Cordillera, Mixedwood Plains	
**Superfamily Bostrichoidea**						
Bostrichidae	24^11^	24 (4)	13	1–2	Taiga ecozones and southward	
Dermestidae	39	47 (16)	29	3–5	southern Arctic and southward	
Endecatomidae	?^11^	1	0	0	Prairies, Boreal Shield, Mixedwood Plains, Atlantic Maritime	
Ptinidae	85^12^	99 (19)	64	6–9	Taiga ecozones and southward	Webster et al. 2016b
**Superfamily Lymexyloidea**						
Lymexylidae	1	1	1	0	Boreal Shield, Boreal Plains, Mixedwood Plains, Atlantic Maritime	
**Superfamily Tenebrionoidea**						
Aderidae ^13^	8	11 (2)	11	1	Boreal Shield and southwards in the east, south of boreal in the west	Barber and Bouchard 2017
Anthicidae	25	65 (3)	33	12	Taiga ecozones and southward	
Boridae	?^14^	2	2	0	Taiga ecozones and southward	
Ciidae	23	29 (1)	25	0	Taiga ecozones and southward	
**Superfamily Tenebrionoidea**						
Ischaliidae	?^15^	2	2	0	Montane Cordillera, Western Interior Basin, Boreal Shield, Mixedwood Plains, Atlantic Maritime, Pacific Maritime	
Melandryidae	35^16,19^	43	40	0	Taiga ecozones and southward	
Meloidae	40	46	34	0	Boreal ecozones and southward	
Mordellidae	50^17^	75	107	42	Boreal ecozones and southward	
Mycetophagidae	10	16 (2)	15	0	Boreal ecozones and southward	
Mycteridae	2	4	2	0	Montane Cordillera, Mixedwood Plains, Atlantic Maritime	
Oedemeridae	15	13 (1)	7	0	Boreal ecozones and southward	
Prostomidae	1	1	0	0	Pacific Maritime	
Pyrochroidae	21^15^	21	15	0	Boreal ecozones and southward	
Pythidae	?^14^	6	9	3	Boreal ecozones and southward	
Ripiphoridae	7	11	2	0	Boreal Shield and southwards in the east, south of boreal in the west	
Salpingidae	10^14^	10 (1)	9	0	Taiga ecozones and southward	
Scraptiidae	?^14,17^	20	48	30	Boreal ecozones and southward	
Stenotrachelidae ^18^	6	9	6	0	Boreal ecozones and southward	
Synchroidae	?^19^	2	1	0	Boreal Shield, Prairies, Mixedwood Plains, Atlantic Maritime	
Tenebrionidae	106^20^	137 (15)	92	10	Taiga ecozones and southward	Bousquet et al. 2018
Tetratomidae	8	20 (1)	16	0	Boreal ecozones and southward	
Zopheridae	23^21,22^	19 (1)	6	0	Boreal ecozones and southward	
**Superfamily Cleroidea**						
Biphyllidae	1	1	0	2	Mixedwood Plains, Atlantic Maritime	
Byturidae	2	1	1	0	Boreal ecozones and southward	
Cleridae	40	52 (6)	36	3	Boreal ecozones and southward	
Melyridae	30	53 (2)	46	0	Boreal ecozones and southward	
Trogossitidae	23	22	4	0	Taiga ecozones and southward	
**Superfamily Cucujoidea**						
Cucujidae	25^24^	8	6	0	Taiga ecozones and southward	
Cryptophagidae	45	68 (10)	71	5	Taiga ecozones and southward	
Cybocephalidae	?^25^	1	4	3	Montane Cordillera, Prairies, Mixedwood Plains	
Erotylidae	17	28 (1)	20	0	Boreal ecozones and southward	
Kateretidae	?^25^	8 (2)	5	2	Taiga ecozones and southward	
Laemophloeidae	?^24^	13 (3)	13	0	Boreal Shield and southwards in the east, south of boreal in the west	
Monotomidae ^26^	15	27 (5)	13	6	Montane Cordillera, Western Interior Basin, Mixedwood Plains, Atlantic Maritime, Pacific Maritime	
Nitidulidae	95^25^	99 (11)	78	12	Taiga ecozones and southward	Webster et al. 2016c
Passandridae	?^24^	1	1	0	Mixedwood Plains	
Phalacridae	10	8	19	11	Boreal Shield and southwards in the east, south of boreal in the west	
**Superfamily Cucujoidea**						
Sphindidae	3	6	5	0	south of Boreal ecozones	
Silvanidae	?^23^	16 (6)	8	0	Boreal ecozones and southwards	
**Superfamily Coccinelloidea**						
Anamorphidae	?^26^	2 (1)	2	0	Mixedwood Plains, Atlantic Maritime	
Bothrideridae	?^22^	3	0	0	Montane Cordillera, Western Interior Basin, Pacific Maritime, Mixedwood Plains	
Cerylonidae	6^27^	4	5	1	Boreal ecozones and southward	
Coccinellidae	120	162 (10)	136	0	Taiga ecozones and southward	Ratzlaff et al. 2016
Corylophidae	5	16 (2)	24	8	Boreal ecozones and southward	
Endomychidae	10^26^	13	9	1	Boreal ecozones and southward	
Euxestidae	?^27^	2	0	0	Mixedwood Plains, Atlantic Maritime	
Latridiidae	45	60 (21)	83	23	Taiga ecozones and southward	
Mycetaeidae	?^26^	1 (1)	0	0	Boreal Shield, Mixedwood Plains, Atlantic Maritime, Pacific Maritime	
Murmidiidae	?^27^	2 (1)	1	0	Boreal Shield, Mixedwood Plains	
Teredidae	?^22^	1	0	0	Pacific Maritime	
**Superfamily Curculionoidea**						
Anthribidae	18	22 (1)	20	2	Boreal ecozones and southward	Webster et al. 2016c
Attelabidae	?^28^	14	14	2	Boreal Shield and southwards in the east, south of boreal in the west	
Brachyceridae	?^28^	18 (2)	10	0	Taiga ecozones and southward	
Brentidae	?^28^	48 (8)	54	15	Taiga ecozones and southward	
Cimberididae ^29^	4	8	7	0	Boreal ecozones and southward	
Curculionidae	1113^28^	826 (107)	433	75	southern Arctic and southward	Webster et al. 2016c, de Tonnancour et al. 2017
Dryophthoridae	?^28^	27 (3)	10	0	Boreal ecozones and southward	
**Superfamily Chrysomeloidea**						
Cerambycidae	350	375 (9)	306	10–30	southern Arctic and southward	Bousquet et al. 2017
Chrysomelidae	588^30^	595 (56)	339	40–170	southern Arctic and southward	Marshall and Paiero 2016
Megalopodidae	?^30^	7 (1)	4	0	Boreal Shield and southwards in the east, south of boreal in the west	
Orsodacnidae	?^30^	1	1	0	Boreal Shield and southwards in the east, south of boreal in the west	
**Total**	**6742**	**8302 (639)**	**5750**	**1078−1284**		

**^1^**Classification following Bousquet et al. (2013) with updates from Short and Fikáček (2013), Robertson et al. (2015), Cline et al. (2014), Shin et al. (2018). **^2^**Current Canadian richness based on Bousquet et al. (2013) with updates from the literature indicated under ‘Information sources’, the number in parentheses represents the number of non-native species included in the total. **^3^**Barcode Index Number, as defined in Ratnasingham and Hebert (2013).

**^4–30^**Family-level classification in Campbell et al. (1979) differing from the present study includes: **^4^**Rhysodidae and Trachypachidae as Carabidae; **^5^**Scirtidae as former Helodidae; **^6^**Helophoridae and Hydrochidae as Hydrophilidae; **^7^**Agyrtidae as Silphidae; **^8^**former Leptinidae treated separately; **^9^**former Pselaphidae, Scydmaenidae, Micropeplidae and Scaphidiidae treated separately; **^10^**all scarabaeoid families except Lucanidae and Passalidae treated as Scarabaeidae; **^11^**Endecatomidae as Bostrichidae and former Lyctidae treated separately; **^12^**former Anobiidae treated separately; **^13^**Aderidae as former Euglenidae; **^14^**Boridae and Pythidae treated as Salpingidae; **^15^**former Pedilidae treated separately and Ischaliidae as Pyrochroidae; **^16^**some Scraptiidae as Melandryidae; **^17^**some Scraptiidae as Mordellidae ; **^18^**Stenotrachelidae as former Cephaloidae; **^19^**Synchroidae as Melandryidae; **^20^**former Alleculidae and Lagriidae treated separately; **^21^**former Colydiidae treated separately; **^22^**Bothrideridae and Teredidae as former Colydiidae; **^23^**Laemophloeidae, Passandridae and Silvanidae as Cucujidae; **^24^**Kateretidae and Cybocephalidae as Nitidulidae; **^25^**Monotomidae as former Rhizophagidae; **^26^**Anamorphidae and Mycetaeidae as Endomychidae; **^27^**Euxestidae and Murmidiidae as Cerylonidae; **^28^**former Platypodidae and Scolytidae treated separately, and Attelabidae, Brachyceridae, Apioninae (Brentidae) and Dryophthoridae as Curculionidae; **^29^**Cimberididae as subfamily Cimberidinae of Nemonychidae; **^30^**Megalopodidae and Orsodacnidae as Chrysomelidae, and former Bruchidae treated separately.

**Reconciling BINs with morphological data – a Canadian example**

Barcode reference libraries for beetles and bees in taxonomically well-studied Central Europe (Hendrich et al. 2014, Schmidt et al. 2015) show that most BIN clusters are highly congruent with taxa already recognised by science. There, most discordance between BINs and recognised taxa likely reflects cases of unrecognised species diversity or species pairs with very similar COI sequences that, while considered one BIN due to shallow divergence, still clustered into species (Hendrich et al. 2014). However, it will be important to assess whether BINs closely approximate real taxa in other regions. We anticipate that barcoding will be similarly effective for characterising the Canadian fauna since both regions are climatologically and topographically similar with shared glaciation history, and with many beetle genera in common.

One DNA barcoding-related discovery is a cryptic species of burying beetle (Silphidae: *Nicrophorus* Fabricius) that was discovered in North America based on congruent evidence from ecological data, mating studies, morphology and DNA barcode data (Sikes et al. 2016). *Nicrophorus* is considered taxonomically very well studied in North America but the cryptic lineage was first highlighted by a different BIN than specimens from the Palaearctic and Alaska (Sikes et al. 2016). We anticipate that the taxonomic integration of DNA barcode data will provide many other insights about the Canadian fauna. The pressing need to carefully and authoritatively link Linnaean taxonomy with molecular reference databases such as BOLD through taxonomic research was stressed by Somervuo et al. (2017) and is re-emphasised here. This need was recently recognised in Canada, with over 5000 beetle species (summarised by Bouchard et al. 2017) added to BOLD in recent years. Although a general, species-focused analysis of the Canadian beetle fauna (similar to Hendrich et al. 2014) is premature, it is possible to examine the congruence of BINs with the taxonomy of a group of well-revised but diverse beetles.

We can partially test BIN congruence using the subtribe Quediina (sensu Brunke et al. 2016), a diverse lineage of rove beetles (Staphylinidae) and the subject of modern taxonomic revision in North America, including critical examination of male genitalia for species concepts (Smetana 1971a, b, 1973, 1976, 1978, 1981, 1990). They are generalist predators, may be important predators of pest insects and are often abundant in decaying organic matter (Smetana 1971a). Currently, 64 species of *Quedius* and *Quedionuchus* are recorded from Canada (Bousquet et al. 2013) and of these, 42 (66%) are represented in BOLD by sequences of authoritatively identified specimens. A total of 52 BINs represent Canadian Quediina in BOLD and most BIN incongruence with existing taxonomy is due to unrecognised species diversity. Although four ‘well-known’ species are currently considered Holarctic in distribution, Nearctic specimens form separate BINs from their Palaearctic counterparts in three of these. One Canadian *Quedius* has two traditionally recognised subspecies for which BINs are 6% divergent and will likely be considered morphologically diagnosable species. Another four BINs correspond to still unidentified species and further work is needed to discern whether they belong to described or undescribed taxa. Two Palaearctic *Quedius* species appear present but unverified and unreported from Canada. Four cases exist where a valid species of *Quedius* contains two BINs that do not correspond to morphological differences. In these cases, BOLD may have oversplit species due to algorithm artifacts based on material limited in number and geographic coverage, and BINs may be later combined in BOLD when additional sequences are included. Taxonomic research involving these putative lineages, including study of type specimens for available names, is in progress. No described species of Quediina shared BINs with any other species, indicating that the species are not ‘oversplit’, likely due to informative variation in male genitalia. Thus, only 7.7% of BINs were incongruent with species level clusters after cases of unrecognised species diversity were removed (a further 11.5%). A similar result was found for the well-studied Quediina of Central Europe where similar diversity (71 species, Assing and Schülke 2012) was represented by about 51% coverage (36 species with 39 BINs) in BOLD and only 7.7% of BINs (involving two species) were incongruent with prevailing species concepts (Hendrich et al. 2014).

The utility of BOLD as a proxy for biodiversity should be demonstrated over a broader taxonomic and geographic scale (Bergsten et al. 2012), as the BOLD database is highly skewed toward Canadian specimens. However, it is promising that gaps in the variation of male genitalia of rove beetles, typically used by taxonomists, correspond remarkably well to gaps in sequence variation identified by BINs. This suggests that BINs may provide a proxy for beetle diversity in North America and could be useful for highlighting taxonomic groups needing research attention (as done above by family).

**Future priorities**

The number of known species from Canada will continue to increase as new species are described, new populations of described species are discovered and species arrive as a result of climate change or global trade. While further taxonomic work on Staphylinoidea, Cucujoidea, Chrysomeloidea and Curculionoidea will continue to add many species to the Canadian fauna, new biosystematics work on several poorly studied families (e.g., Mordellidae, Scraptiidae, Latridiidae, Ptiliidae, and Scirtidae) is greatly needed. Although recent collecting in eastern Canada has yielded many discoveries, these biomes and, especially, those of central and western Canadian provinces remain inadequately sampled. A renewed effort toward exploring the Canadian beetle fauna will be critical for the documentation of the more than 1000 unrecorded or undescribed species that are estimated to be undetected or undescribed in Canada. Since DNA barcoding is a useful tool for assessing species diversity and appears to be highly compatible and synergistic with traditional morphological taxonomy, knowledge of beetle diversity in Canada will further benefit from continued development of the DNA barcode library through focused collecting, authoritative vouchering and continued integrative taxonomic research. However, improved and continued documentation of the Canadian fauna can only be achieved with new funding for surveys, including a variety of sampling methods, and by hiring or otherwise supporting scientists that include taxonomic work on the Canadian fauna as part of their research profile.
